# Reviews and Book Notices

**Published:** 1883-04

**Authors:** 


### Reviews and Book Notices.

---

ARTICLE XV.—PHYSICAL EXPLORATION OF THE LUNGS BY MEANS OF AUSCULTATIOM AND PERCUSSION. Three Lectures by AUSTIN FLINT, M.D., Delivered before the Philadelphia County Medical Society.

This is a reprint in book form of these lectures, which were delivered by the well-known teacher and author at the invitation of the Philadelphia County Medical Society. The lectures have recently appeared in one of the Eastern journals. Although to many the subject is old, there is still a large portion of the medical profession who are not entirely conversant with the subject of physical diagnosis, and to them these lectures cannot fail to be of much interest. Compliments are unnecessary when this author's name is found. E. F. I.

